# Development of interstitial cells of Cajal in the human digestive tract as the result of reciprocal induction of mesenchymal and neural crest cells

**DOI:** 10.1111/jcmm.13375

**Published:** 2017-11-28

**Authors:** Goran Radenkovic, Dina Radenkovic, Aleksandra Velickov

**Affiliations:** ^1^ Department of Histology and Embryology Faculty of Medicine University of Nis Nis Serbia; ^2^ UCL Medical School University College London (UCL) London UK

**Keywords:** interstitial cells of Cajal, neural crest cells, enteric nervous system, human, digestive tract

## Abstract

Neural crest cells (NCC) can migrate into different parts of the body and express their strong inductive potential. In addition, they are multipotent and are able to differentiate into various cell types with diverse functions. In the primitive gut, NCC induce differentiation of muscular structures and interstitial cells of Cajal (ICC), and they themselves differentiate into the elements of the enteric nervous system (ENS), neurons and glial cells. ICC develop by way of mesenchymal cell differentiation in the outer parts of the primitive gut wall around the myenteric plexus (MP) ganglia, with the exception of colon, where they appear simultaneously also at the submucosal border of the circular muscular layer around the submucosal plexus (SMP) ganglia. However, in a complex process of reciprocal induction of NCC and local mesenchyma, c‐kit positive precursors are the first to differentiate, representing probably the common precursors of ICC and smooth muscle cells (SMC). C‐kit positive precursors could represent a key impact factor regarding the final differentiation of NCC into neurons and glial cells with neurons subsequently excreting stem cell factor (SCF) and other signalling molecules. Under the impact of SCF, a portion of c‐kit positive precursors lying immediately around the ganglia differentiate into ICC, while the rest differentiate into SMC.

**Table:** 

• **Introduction**
• **Development of the ENS**
• **Interstitial cells of Cajal**
• **Differentiation of c‐kit positive cells in the human digestive tract**
• **Differentiation of ICC from c‐kit positive cells**
• **Conclusion**
• **Acknowledgements**
• **Conflict of interests**

## Introduction

NCC migrate into different parts of the body and express their strong inductive potential (inducing differentiation of numerous structures) and multipotency (they themselves differentiate into various cell types with diverse functions). One of the sites of NCC migration is the digestive tube, where the cells from two different neural crest segments, vagal and sacral, can be found 1, 2, 3, 4. In the digestive tube, NCC induce differentiation of muscular structures and ICC along a rostrocaudal gradient, and they themselves differentiate into neurons and glial cells of the ENS 5, 6, 7, 8. Smooth muscle cells, ICC and enteric neurons are required for the onset of peristalsis, a prerequisite for normal bowel function. Some authors have suggested another possible migration wave of neural tube cells—the ventrally emigrating neural tube (VENT) cells into the foregut. Foregut is a part of primitive gut giving rise to the oesophagus, stomach and first part of duodenum. It is thought that VENT cells are able to differentiate into nerve, glial cells and intramuscular subtypes of ICC in these portions of the digestive tube 9, 10. Our hypothesis is that NCC induced mesenchymal cell differentiation into ICC, but in the manner that mesenchymal cells differentiated first into c‐kit positive precursors common for both ICC and SMC. C‐kit positive precursors could have an impact on the guidance of NCC migration, as well as on their final differentiation into neurons and glial cells and formation of myenteric ganglia. Neurons are one of the sources of SCF 11, 12 and other signalling molecules, determining differentiation of c‐kit positive precursors. Recent data have shown that besides the aforementioned Kit signalling pathway, there are additional signalling pathways which play a role in the differentiation and proliferation of ICC (neuronally derived nitric oxide, serotonin signalling through the 5‐HT2B receptor, interleukin 9, insulin and IGF‐1 signalling through stem cell factor) 13, 14, 15, 16. Under the influence of SCF, a portion of c‐kit positive precursors lying immediately around the ganglia differentiate into ICC, while the rest differentiate into SMC.

## Development of the ENS

Most of the ENS develops from the vagal segment that arise at the level of somites 1–7, while the sacral (that lies caudally to somite 28) contributes to the ENS along the postumbilical gut 2, 17, 18, 19, 20. Vagal NCC leave NC at week 4 and populate the region of pharyngeal arches. They enter the posterior wall of the anterior gut, surrounding it and continue their rostrocaudal migration route 20, 21. NCC migrate into the external portion of the gut wall 6, 7, immediately beneath the serosa, in the form of an uninterrupted chain of cells that continues in the caudal direction 22, 23, 24. On their way, NCC intensely divide, interacting with the surrounding cells and differentiate into neurons and glial cells of the MP 7, 25, 26, 27. It has been proposed that NCC transit through several phenotypes before reaching the mature neuron form 28, with frontal cells retaining the NCC phenotype. These frontal cells coexpress Sox 10, RET, p75 and Phox2b, a NCC markers, but they do not express neuronal markers, confirming the assumption that the migrating frontal cells have not commenced their differentiation into neurons 23, 28, 29, 30, 31. Behind the undifferentiated NCC, cells are at different stages of differentiation, with neurons appearing before glial cells 28, 32. Several studies have shown that when NCC differentiate into neurons, they lose their migratory potential 33, 34.

The local mesoderm excretes a number of factors which primarily secure the survival of NCC, then prolong their proliferation and support their migration along the digestive tube; above all, these are GDNF (glial cell line‐derived neurotrophic factor), Endothelin‐3 (ET‐3), BMP2/4 and others 35, 36, 37, 38. Mesenchymal cells thus postpone differentiation of NCC into ganglia and enable their propagation along the primitive gut.

NCC contain the RET receptor 31, 39, 40, 41, mediating local mesodermal influences *via* GDNF production 26, 42, 43, providing thus the proliferation of frontal NCC. In particular, GDNF is not only a mitogenic, but also a chemotactic factor, determining rostrocaudal continuation of the NCC chain 44, 45, 46, 47. The wave of maximum GDNF expression shifts rostrocaudally and pulls along the tip of the NCC chain made of newly proliferated cells with the highest migratory potentials 33, 34, 48. Because GDNF attracts NCC, it is possible that the GDNF gradient is important in leading the advance of NCC down the gut 48.

A body of experimental data shows that neurons do not migrate, but that the majority of immature neurons are capable of migrating, although slower and at shorter distances compared to NCC 49. Neuroblasts are situated within the chain formed by migrating NCC 30, 32, 50, and they probably first slow down the migration and at a later stage fall behind and group together to form ganglia 51, 52. After a MP is formed, 2 or 3 weeks later, the ganglia of the SMP are formed in the human digestive tract. They develop during the secondary migration wave from the cells that migrate centripetally from the MP region 53, 54. The development of ENS in the oesophagus, stomach and small bowel follows this pattern, while the development in the large bowel is different. On their route through the digestive tube, migrating NCC stay in the caecum for a while and then continue their migration through the large bowel, following a somewhat different route 55. The reason for this arrest of NCC in the region of caecum is not known 56. It should be stressed that caecum has been the place of maximum GDNF and ET‐3 expression 43, 57, 58. While they migrate to the caecum in the form of an uninterrupted chain of cells localized in the outer portion of the wall, immediately beneath the serosa 23, 24, in the caecum and later in the colon, they continue their migration as individual, isolated cells that occassionally group together 55. A significant difference regarding the ENS development in the large bowel is also the fact that the cells of the sacral neural crest segment also contributes to the enteric neurons and glial cells of both the myenteric and the submucosal plexuses; sacral NCC migrate in the caudorostral direction and meet the vagal NCC 2, 22, 59, 60, 61.

## Interstitial cells of Cajal

ICC are specialized network‐forming cells distributed within and around the smooth muscle wall of the digestive tract, capable of generating and propagating the electric slow waves 62, 63, 64. In addition to their pacemaker role, ICC are implicated in enteric neurotransmission and acting as stretch receptors in the gastrointestinal tract 65, 66, 67, 68. It has been shown that there are several ICC subtypes depending on their anatomical locations, morphologic and functional criteria as follows: ICC lying between the circular and longitudinal muscle layer and around the MP ganglia (termed the ICC‐MP subtype), ICC located in muscle bundles, between muscle cells (the ICC‐IM subtype), ICC situated along the submucosal margin of the circular muscle layer (the ICC‐SM subtype), ICC lying within the connective tissue septa which surround bundles of the muscle (the ICC‐SEP subtype) and ICC located in the small intestine at the level of the deep muscular plexus (the ICC‐DMP subtype). Throughout the digestive tube, the ICC lying around the MP ganglia (termed the ICC‐MP subtype) play the pacemaker role 62, 63, 69; only in the colon, in addition to ICC‐MP, the ICC‐SMP play the pacemaker role as well 70, 71, 72. Other ICC subtypes are functionally intercalated between the ENS and SMC 66, 73 or they function as mechanoreceptors 74, 75. Although it has been thought in the past that ICC represent a kind of neuron, it has been later reliably established that they are mesenchymal by origin 12, 30, 76, 77. ICC express the gene product of *c‐kit,* a proto‐oncogene that encodes the receptor tyrosine kinase. Stimulation of the Kit receptor (the natural ligand of which is SCF) is essential for their differentiation and survival 62, 78, 79. Most ICC subtypes, ICC‐MP and ICC‐SM included can be identified by labelling with c‐Kit antibody 80, 81. This fact has markedly facilitated ICC identification and made possible the study of their appearance in the wall of the digestive tube. In addition, VENT cells as well can have a role in the development of particular ICC subtypes (ICC‐IM) in the oesophagus, stomach and the first part of duodenum, portions of the digestive tube that arise from the foregut 9, 10.

## Differentiation of c‐kit positive cells in the human digestive tract

During the development of human digestive tract, c‐kit positive cells, morphologically different from mature ICC, appear at the end of the embryonal period. C‐kit positive cells appear firstly in the oesophagus and stomach, then in the small bowel and finally in the large bowel 55, 82, 83, 84, 85. C‐kit positive cells emerge along the digestive tube following the rostrocaudal gradient, in the same way, NCC colonizing the digestive tube. What is important is that c‐kit positive cells appear just at the level of the NCC migration route, immediately around the chain formed by NCC during their migration along the digestive tube.

**Figure 1:**
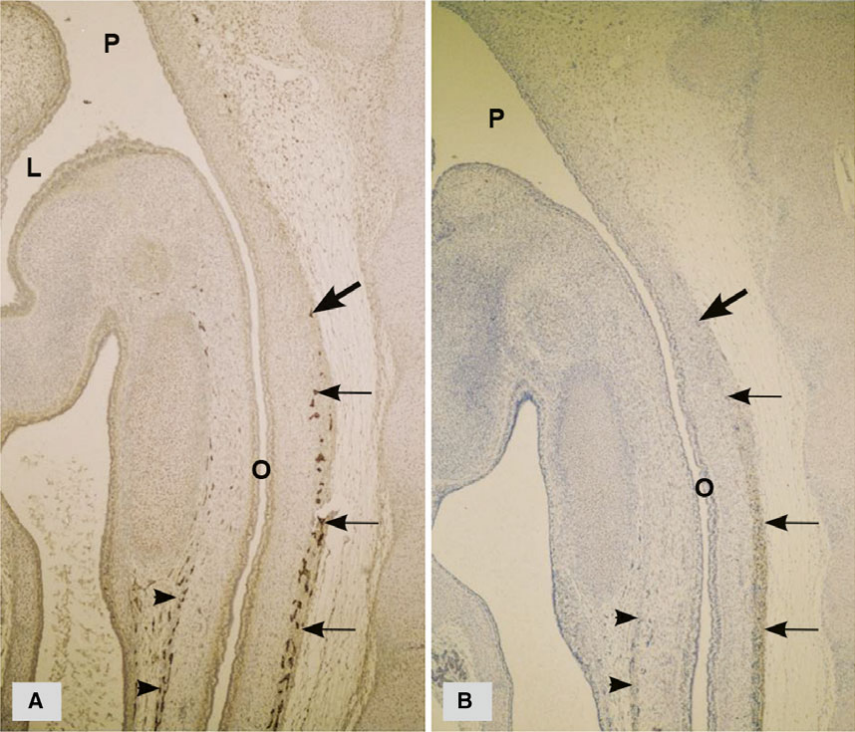
Sagittal sections of a human embryo at 8 weeks of development (two very close sections). (**A**) PGP9,5—immunohistochemistry; the inceptions of MP ganglia are numerous in the posterior wall (arrows) and slightly less abundant in the anterior of the primitive oesophagus (arrow heads). (**B**) c‐kit immunohistochemistry; c‐kit positive cells in the posterior (arrows) and anterior wall of the primitive oesophagus (arrow heads) form a wide belt of cells around the inceptions of MP ganglia. Large arrows indicate the spot from which MP ganglia (**A**) and c‐kit positive cells (**B**) started to differentiate, as both differentiate along the digestive tube following the rostrocaudal gradient. O, oesophagus; P, pharynx; L, larynx. (Radenkovic, unpublished data).

Consecutive longitudinal sections of the human embryonal oesophagus (Fig. 1A, B) showed that c‐kit positive cells were present exactly at the spot where the differentiation of the MP ganglia inception started and distally from that spot, that is, in the direction of NCC migration along the digestive tube. The inception of MP ganglia (Fig. 1A) and c‐kit positive cells (Fig. 1B) started to differentiate from the same proximal spots located in the posterior wall of the oesophagus. These c‐kit positive cells are much more abundant and morphologically different from the described mature ICC 82, 83, 84. C‐kit positive cells are very similar to blast cells, with a small pleomorphic body, containing a large nucleus and numerous but short cellular processes. They form a wide belt of cells in the outer portion of the wall of the oesophagus, stomach and first part of the duodenum and surround the NCC in the chain that represent future MP ganglia 55, 82, 83, 84. The environment is thus changed for all NCC in the chain of migration except for the frontal group of cells (newly proliferated NCC cells), which are the only ones remaining in direct contact with mesenchymal cells. Exactly at the front of the chain, there is the spot of maximum GDNF expression 43, 86 and that maximum shifts in the rostrocaudal direction following the prolonged migration wave. C‐kit positive cells probably reduce GDNF production and as they surround the NCC chain on the sides, they act as a kind of ‘funnel’ that orients chain continuation in the caudal direction. The cells in the chain slow down their migration and start to differentiate into neurons and glial cells and group together into inception of MP ganglia.

The appearance of c‐kit positive cells in the oesophagus, stomach and first part of duodenum follows this pattern 82, 83, 84. In the rest of the small bowel, all the way to the caecum, c‐kit positive cells appear in a similar way, although in the form of a very narrow chain of cells lying in the outer portion of the wall, immediately beneath the serosa, around the migrating NCC, that is inception of MP ganglia 84, 85, 87, 88. The difference in the appearance of c‐kit positive cells in the oesophagus, stomach and the first part of duodenum compared to the rest of the small bowel could perhaps be explained by the presence of VENT cells which populate only the foregut 9, 10. VENT cells could potentially contribute to that larger number of c‐kit positive cells observed in the organs developing from the foregut. A reduced number of c‐kit positive cells along the small intestine may be a result of the diminished proliferative capacity of NCC 47. However, this assumption requires confirmation by future studies.

**Figure 2:**
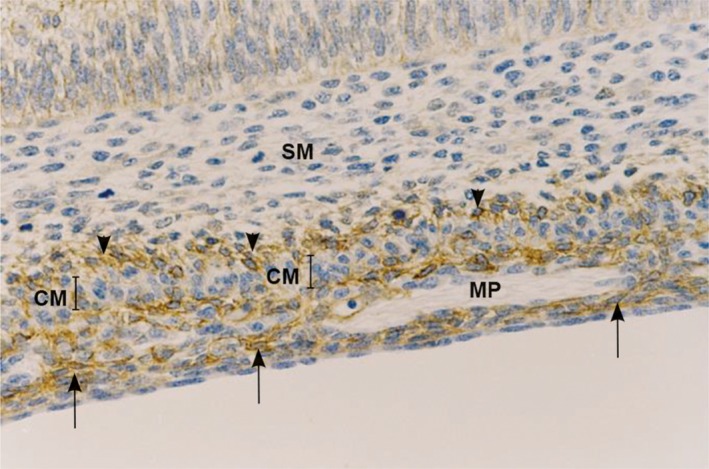
Longitudinal section of the proximal human colon at 9–10 weeks. C‐kit immunohistochemistry; c‐kit positive cells are present in the form of two parallel bands of cells: beneath the serosa, around the inception of MP ganglia (arrows) and at the submucosal border of the circular muscular layer, around the inception of SMP ganglia (arrow heads). MP, myenteric plexus; SM, submucosa; CM, circular muscle.

The observed difference in the appearance of c‐kit positive cells in the large bowel is significant. In particular, c‐kit positive cells appear in the form of two parallel belts of cells, the first being situated around the inception of MP ganglia, and the second at the submucosal border of the circular muscle layer, around the inception og SMP ganglia 85, 89 (Fig. 2).

The reason for their simultaneous two‐level differentiation can be explained by the differences in the manner of migration of NCC in the colon compared to the rest of the digestive tube 55, as described in the previous chapter. Vagal NCC are scattered widely throughout the mesenchyme of the wall of the colon, and they migrate in a wide wave that covers almost the entire width of the wall from serosa to submucosa, although the presumptive submucosal region was sparsely populated 86, 89. Such a distribution is possible as muscle layers are still undifferentiated in the colon. We should be aware of the fact that in the colon, the cells from the sacral neural crest segment are involved in the development of ENS 59, 60, which may have an impact on the pattern of differentiation of c‐kit positive cells. A recent study has shown an additional pathway for NCC migration to the colon. Due to close proximity of the midgut and hindgut, some NCC pass directly from the midgut to the hindgut as isolated cells *via* the mesentery 90. We may say that the process of differentiation of c‐kit positive cells and their feedback impact on the slowing down of migration, differentiation and grouping of NCC into inception of ganglia takes place simultaneously at two levels in the colon, in the MP and in SMP regions.

## Differentiation of ICC from c‐kit positive cells

In the weeks of development to follow, the number of c‐kit positive cells is markedly reduced, and the rest of them assume the morphological characteristics of mature ICC, as described in a later foetal period and after birth 82, 83, 84, 85. In the period when the number of c‐kit positive cells is being reduced, SMC of the longitudinal layer appear as well, so it seems possible that a part of c‐kit positive cells differentiate into SMC 84, 91, 92. This hypothesis is corroborated by the papers about ICC transdifferentiation, that is, the fact that in the absence of KIT receptor stimulation ICC can differentiate into SMC as well as the morphological and functional similarities between ICC and SMC 93, 94.

**Figure 3:**
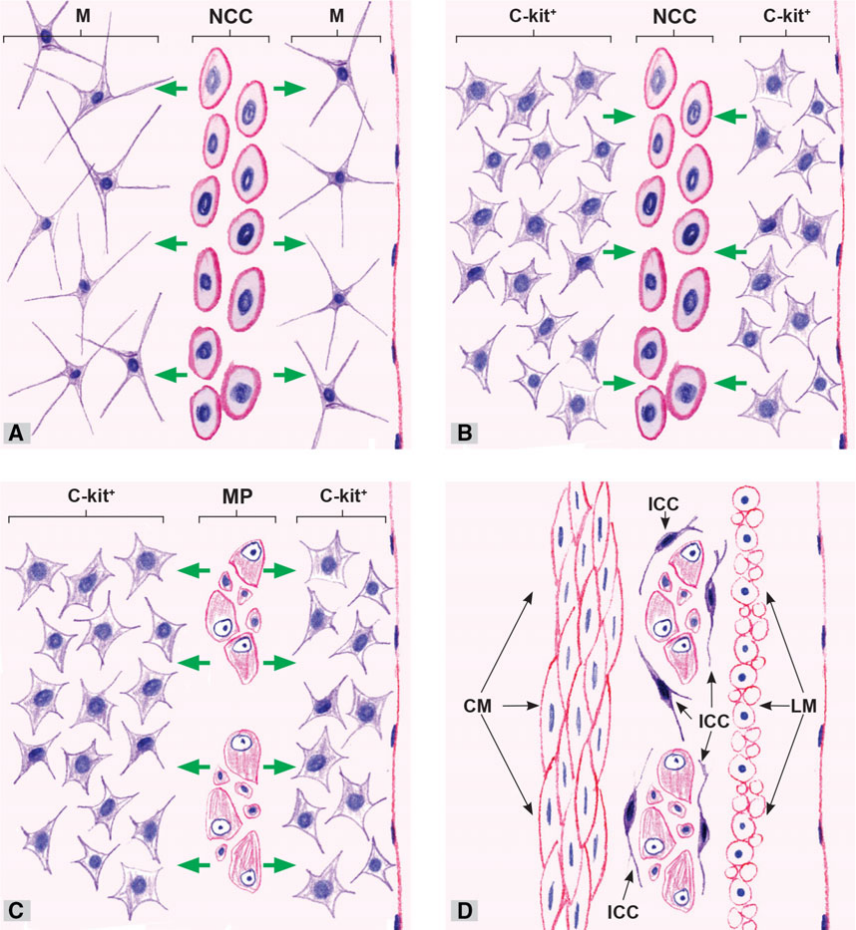
Possible pathways of interaction and reciprocal induction of NCC and mesenchymal cells in the wall of the human primitive gut. (**A**) NCC populate the outer portion of the wall of the primitive gut and under the action of growth factors (GDNF, ET‐3, BMP2/4) excreted by mesenchymal cells they rapidly proliferate. At the same time, NCC induce differentiation of the adjacent mesenchymal cells into c‐kit positive cells. (**B**) c‐kit positive cells probably reduce GDNF production and induce differentiation of NCC into neurons (cells with a neuronal phenotype) and glial cells, which slow down their migration and group to form the inception of MP ganglia. (**C**) Neurons as one of the sources of STF induce differentiation of c‐kit positive cells situated immediately around them into ICC‐MP. (**D**) The rest of c‐kit positive cells differentiate into SMC, mostly of the longitudinal layer. M, mesenchymal cells; NCC, neural crest cells (NCC); c‐kit+, c‐kit positive cells; MP, myenteric plexus; ICC, interstitial cells of Cajal; CM, circular muscle; LM, longitudinal muscle.

The probability exists as well that a some of these c‐kit positive cells differentiate into ICC and specifically into those lying immediately adjacent to the MP ganglia so that ICC‐MP cells are the first to appear in the oesophagus, stomach and small bowel [55, 82, 83, 84, 85, 88, 95]. They lie at the MP ganglia borders (Fig. 3), surrounding it entirely with their bodies and processes, but they do not present within the ganglia. Experimental data showed that enteric neurons express SCF 11, 12, 96. Expression of SCF by neurons might induce differentiation of c‐kit positive cells situated immediately around them into ICC‐MP. Taking into consideration the hypothesis by Huizinga and White, the above‐described c‐kit positive cells represent ICC precursors, which are capable of differentiating into ICC (depending on the Kit receptor stimulation), or in absence of such stimulation, into SMC 97. Recent data suggest that in the adult gut, there are precursor/stem cells the differentiation of which can produce not only ICC but also SMC and perhaps other cell types involved in the maintenance of ICC network 98. Chen *et all*. have proposed the hypothesis that some c‐kit positive cells maintain the characteristics of ICC progenitor cells after birth as well, thus being able to differentiate into ICC if there is a need 99. Recent data have shown that bone marrow‐derived Kit+ cells are able to repopulate the small intestine in response to intestinal injury 100. After MP ganglia differentiation, the neurons migrate centripetally through the circular layer to the submucosal border where they differentiate into SMP so that SMP appears 2–3 weeks after MP. ICC‐SMP appear 2–3 weeks after diferentiation of ICC‐MP 53, 55, 82, 83. This occurs throughout the digestive tube except in the colon, where ICC differentiation follows a different pattern. ICC‐MP and ICC‐SMP appear simultaneously in the colon, and the reason for this is that c‐kit positive cells simultaneously differentiate at the MP and SMP levels. As previously stated in the chapter about ICC, in all portions of the digestive tube, the ICC with pacemaker role 73, 101, 102 are the first to differentiate in all portions of the human digestive tube.

During the NCC migration along the digestive tube, they induce differentiation of mesenchymal cell into c‐kit positive precursors. These precursors perhaps induce NCC differentiation into neurons and glial cells. The impact of c‐kit positive precursors on NCC cannot be definitely confirmed at the moment, but experimental findings have indicated the possibility. Zhao *et all*. demonstrated *in vitro* that ICC can promote the differentiation of neuroepithelial stem cells into neurons 103. These observations suggest that c‐kit positive precursors can affect the differentiation of NCC into mature neurons during the development of the human ENS.

## Conclusion

In conclusion, NCC have a decisive impact on the differentiation of ICC in the human digestive tract. Nevertheless, in a complex process of reciprocal induction of NCC and local mesenchyma, c‐kit positive precursors are the first to differentiate, representing probably the common precursors of ICC and SMC. These precursors could have an impact on NCC differentiation into neurons and glial cells. Finally, neurons induce differentiation of c‐kit positive precursors lying immediately around them into mature ICC, while the remaining precursors, in an absence of KIT stimulation, differentiate into SMC.

## Conflict of interest

The authors declare that they have no conflict of interests.
